# Early Administration of Anti-SARS-CoV-2 Monoclonal Antibodies Prevents Severe COVID-19 in Kidney Transplant Patients

**DOI:** 10.1016/j.ekir.2022.08.014

**Published:** 2022-08-30

**Authors:** Juliette Gueguen, Philippe Gatault

**Affiliations:** 1 Service Néphrologie, Hypertension Artérielle, Dialyses et Transplantation Rénale, Tours Laboratoire T2I (Transplantation, Immunologie, Inflammation), France; 2 Hôpital Bretonneau, Tours, France

To the Editor:

We would like to respond to the letter submitted by Pathum Sookaromdee *et al.*^1^ regarding our manuscript entitled “Early administration of Anti-SARS-CoV-2 Monoclonal Antibodies Prevents Severe Covid-19 in Kidney-Transplant Patients”^2^ and potential confounding factors that may have biased our observation.

First, the authors suggest that the course of COVID-19 might be different and thereby the benefit obtained regarding the use of monoclonal antibody might be different between individuals. We agree with this comment. It is likely that clinicians selected patients that they considered having a significant risk of severe COVID-19 during the study period, according to their age and underlying comorbidities. Consequently, we used propensity score matching to ensure comparability between the patients who received monoclonal antibody and those who did not. In our opinion, the most relevant question remains whether our results support the use of monoclonal antibody in patients with the lowest risk of worsening COVID-19, especially “young” renal transplant patients without comorbidities. Because the safety profile of monoclonal antibody is excellent and reassuring, this question raises mainly economic pain. It will be necessary to assess specifically this point. To determine whether monoclonal antibody mitigate the mild COVID-19 and decrease indirect cost such as medical leave would be very interesting.

Second, the authors remind readers that there is a rapid change of epidemiology of the SARS CoV2. This aspect has already been discussed in the discussion part of our paper. Our paper is in regards to the period when the B.1.617 variant was predominant and we wanted to highlight the need of an additive prophylaxis to patients who mostly do not respond to vaccination. Today, genetic escape of variants of concerns is the main limit to the effectiveness of monoclonal antibody, in contrast to vaccine, justifying a continuous assessment of monoclonal antibody usefulness. It is clearly the main pitfall of passive preventive strategy.

Third, authors underline the potential role of unknown sensitization due to asymptomatic COVID-19. Based on our experience and taking into account baseline characteristics, we do not believe that asymptomatic COVID-19 had frequently occurred in our patients. In addition, unidentified seropositive patients could be included in both groups. This comment offers the opportunity to insist on the importance of periodic measures of antispike antibodies concentrations and the need to define protective threshold of monoclonal antibodies. Though personalized preventive strategy based on monoclonal antibodies is now used in some nonresponders to vaccine, this point is particularly critical to personalize the administration schedule of monoclonal antibody, bearing in mind that neutralizing threshold can change according to variants.

We hope we have responded to all the queries and we stand available for further remarks.
